# New Strategies for Echocardiographic Evaluation of Left Ventricular Function in a Mouse Model of Long-Term Myocardial Infarction

**DOI:** 10.1371/journal.pone.0041691

**Published:** 2012-07-27

**Authors:** Carolina Benavides-Vallve, David Corbacho, Olalla Iglesias-Garcia, Beatriz Pelacho, Edurne Albiasu, Sara Castaño, Arrate Muñoz-Barrutia, Felipe Prosper, Carlos Ortiz-de-Solorzano

**Affiliations:** 1 Imaging Unit, Fundación para la Investigación Médica Aplicada, University of Navarra, Pamplona, Navarra, Spain; 2 Cardiology Department, Clínica Universidad de Navarra, Pamplona, Navarra, Spain; University of Otago, New Zealand

## Abstract

**Background:**

The aim of this article is to present an optimized acquisition and analysis protocol for the echocardiographic evaluation of left ventricle (LV) remodeling in a mouse model of myocardial infarction (MI).

**Methodology:**

13 female DBA/2J mice underwent permanent occlusion of the left anterior descending (LAD) coronary artery leading to MI. Mice echocardiography was performed using a Vevo 770 (Visualsonics, Canada) before infarction, and 7, 14, 30, 60, 90 and 120 days after LAD ligation. LV systolic function was evaluated using different parameters, including the fractional area change (FAC%) computed in four high-temporal resolution B-mode short axis images taken at different ventricular levels, and in one parasternal long axis. Pulsed wave and tissue Doppler modes were used to evaluate the diastolic function and Tei Index for global cardiac function. The echocardiographic measurements of infarct size were validated histologically using collagen deposition labeled by Sirius red staining. All data was analyzed using Shapiro-Wilk and Student's t-tests.

**Principal Findings:**

Our results reveal LV dilation resulting in marked remodeling an severe systolic dysfunction, starting seven days after MI (LV internal apical diameter, basal = 2.82±0.24, 7d = 3.49±0.42; p<0.001. End-diastolic area, basal = 18.98±1.81, 7d = 22.04±2.11; p<0.001). A strong statistically significant negative correlation exists between the infarct size and long-axis FAC% (r = −0.946; R_2_ = 0.90; p<0.05). Moreover, the measured Tei Index values confirmed significant post-infarction impairment of the global cardiac function (basal = 0.46±0.07, 7d = 0.55±0.08, 14 d = 0.57±0.06, 30 d = 0.54±0.06, 60 d = 0.54±0.07, 90 d = 0.57±0.08; p<0.01).

**Conclusions/Significance:**

In summary, we have performed a complete characterization of LV post-infarction remodeling in a DBA/2J mouse model of MI, using parameters adapted to the particular characteristics of the model In the future, this well characterized model will be used in both investigative and pharmacological studies that require accurate quantitative monitoring of cardiac recovery after myocardial infarction.

## Introduction

Cardiovascular disease, and more specifically myocardial infarction (MI), is the first cause of morbidity and mortality in the world [1],[2],[3]. Left ventricle (LV) remodeling occurs after myocardial infarction as a result of the abrupt loss of contracting cardiomyocytes. Early expansion of the infarct zone is associated with LV dilation caused by the redistribution of the increased regional wall stress to preserve stroke volume [4]. Between one half and one third of patients experience progressive post-infarction dilatation with distortion of ventricular geometry and secondary mitral regurgitation [5].

Quantitative bi-dimensional transthoracic echocardiography is routinely used to characterize the LV remodeling associated with ischemic cardiomyopathies [6],[7]. The traditional echocardiographic measurements recommended for the evaluation of LV remodeling include estimates of LV end-diastolic and end-systolic volumes and LV mass. LV volumes have been demonstrated to predict adverse cardiovascular follow-up events, including recurrent infarction, heart failure, ventricular arrhythmias, and mitral regurgitation [7].

Several murine models of myocardial infarction exist, based on permanent occlusion of the left anterior descending coronary artery (LAD). These models have been used to elucidate mechanisms of myocardial remodeling and provide new insights into the physiology and treatment of cardiovascular disease [8],[9],[10],[11]. To perform reliable measurements of cardiac function in mouse models, high-resolution echocardiography equipment is available, specifically designed for small animal imaging [12]. This is the case of the Vevo 770 (VisualSonics, Toronto, ON), a high-resolution *in vivo* micro-imaging system, which has been used in this study. Ultrasound imaging provides a fast and inexpensive alternative to magnetic resonance imaging [13],[14],[15] when performing longitudinal follow up studies of cardiac remodeling.

The most common parameters used to evaluate the systolic function of the LV are the fractional shortening (FS%), ejection fraction (EF%) and cardiac output (CO) [16]. In the absence of regional wall motion abnormalities, FS% and EF% are predictably related. In mice however, the extent of the typical cavity obliteration and the associated error introduced in the volumetric measurements is far greater than in humans. Consequently, the use of FS% is more appropriate [16]. The FS% and EF% are routinely calculated using the Teichholz method [17], which assumes that the left ventricular cavity can be represented as a 3D ellipsoid of revolution. However, this might not be a reasonable assumption when the LV adopts the complex shapes caused by regional wall motion abnormalities that are common after MI. Therefore, an alternative approach to calculating EF% exists, based on the shape independent Simpson's rule [8],[18],[19], where the LV endocardial border is traced in multiple slices both in systole and diastole, and the volumes are computed from these tracings.

Using the above method, the LV function can also be measured as the percentage of change in left ventricular cross-sectional area between diastole and systole (fractional area change, FAC%), which has been found to correlate well with EF% both in normal and abnormal subjects [20]. The standard method to estimate FAC% uses cross-sectional area short-axis views at different ventricular levels. However, when the infarction affects the ventricular apex, visualizing the infarct area at medium and upper levels becomes difficult and so it is to visualize the entire endocardial border. In those cases, the standard FAC% measurement is hardly representative of the real damage. Instead we propose to use a single parasternal long-axis view, which results in improved visualization.

Cardiologists, beyond the standard systolic dysfunction, are starting to analyze post-infarction diastolic dysfunction, which precedes the depression of systolic function in patients of all ages suffering from both permanent and temporary ischemic cardiomyopathies. The standard approach to diagnose diastolic dysfunction uses a pulsed-wave Doppler scan of transmitral flow, although a variety of other measurements can be used [21],[22]. The accuracy with which those measurements quantify diastolic dysfunction is still open to discussion [23].

Finally, since the isolated analysis of systolic and diastolic mechanisms may not be reflective of overall cardiac dysfunction, the Tei index has been recently introduced. The Tei Index is a simple, reproducible, quantitative estimate of global dysfunction, independent from heart rate and blood pressure levels, characterized by low inter-observer and intra-observer variability [21],[24],[25],[26]. An estimate of global cardiac function can also be obtained using tissue Doppler imaging.

The aim of the present article is to present an optimized echocardiographic quantification of LV remodeling in a mouse model of myocardial infarction. All the above-described parameters have been included and compared in our evaluation, and those that have morphological significance, correlated with histological measurements.

The rest of the article is organized as follows. In Section 2, we briefly summarize our mice model of MI by LAD ligation, the image acquisition protocols and parameters used to evaluate cardiac function. The results are presented in Section 3. A final discussion (Section 4) and conclusions (Section 5) end the article.

## Methods

### Experimental model

Seventeen 8-week-old female DBA/2J mice (Harlan IBERICA S.L. Barcelona, Spain) underwent coronary artery ligation as described in *Pelacho et al.* [27]. Briefly, the animals were anesthetized with 2% isofluorane, intubated, connected to a ventilator for small animals and placed on a heating table in a supine position. A thoracotomy was then practiced at the left fourth intercostal space. Next the pericardium was opened and the left anterior descending coronary artery was ligated using a 7.0 absorbable suture. The pericardial incision was closed in layers with a 6.0 absorbable suture and the skin incision with 6.0 sutures. Finally, the endotracheal tube was removed and spontaneous breathing restored. The animals were kept in a cage, lying on a heating blanket for several hours until recovered from surgery. The survival rate over the course of the experiment was over 75%. A –sham- group made of fourteen 8-week-old female mice that underwent thoracotomy but did not undergo ligation of the coronary artery was used as control of the experimental group.

All experiments were performed in accordance with the principles of laboratory animal care formulated by the National Society for Medical Research and the guide for the care and use of laboratory animals of the Institute of Laboratory Animal Resources (Commission on Life Science, National Research Council). All animal procedures were approved by the University of Navarra Institutional Committee on Care and Use of Laboratory Animals.

### Echocardiographic studies

#### Echocardiography

Echocardiography was performed using a Vevo 770 ultrasound system (Visualsonics, Toronto, Canada) equipped with a real time micro-visualization scan head probe (RMV-707B) working at a frame rate ranging between 110 and 120 frames per sec (fps). The nosepiece-transducer used has a central frequency of 30 MHz, a focal length of 12.7 mm and 55 µm of nominal spatial resolution. The Vevo 770 is equipped with ECG-gated kilohertz visualization software (EKV™), which synthesizes high temporal resolution B-Mode images by combining several ECG-synchronized heart cycles. The EKV image reconstruction software produces B-mode sequences at up to 1000 frames per second.

#### Animal preparation

Mice were anesthetized with isoflurane (Isoflo®, ABBOTT S.A, Madrid, Spain), at a concentration of 4% (induction) and 1.5% (maintenance) in 100% Oxygen. Each animal was placed on a heating table in a supine position with the extremities tied to the table through four electrocardiography leads. The chest was shaved using a chemical hair remover (Veet, Reckitt Benckise, Granollers, Spain). Warmed ultrasound gel (Quick Eco-Gel, Lessa, Barcelona, Spain) was applied to the thorax surface to optimize the visibility of the cardiac chambers. The heart rate (HR) of the animals was recorded immediately before the echocardiographic study.

#### Views and measurements

Echoes were acquired at baseline (before LAD ligation), and 7, 14, 30, 60 and 90 days after LAD ligation. LV remodeling was quantified according to the guidelines and standards of American Society of Echocardiology, the guide to micro-echocardiography study using the Vevo770 [28] and the Vevo 770® Protocol-Based Measurements and Calculations guide, as described in the following paragraphs.

First we quantified the LV structural analysis. To that end the LV diameters at basal (Dbvi), middle (Dmvi) and apical level (Davi), the end-diastolic (Aread) and end-systolic (Areas) areas were measured on a two-dimensional (B-mode) parasternal long-axis view.

The functional analysis of the heart was next evaluated, starting with the LV systolic function that was measured several ways:

The ejection (EF% tei) and shortening (FS%) fractions were calculated from the LV diameters (LVID d, LVID s) measured on an M-mode examination of the LV. To obtain the classical LV M-mode tracing, the M-mode cursor was vertically positioned at a transthoracic parasternal short axis view –visualizing both papillary muscles-.
The normalized mean velocity of circumferential fiber shortening (VcFc) was calculated as the ratio between the shortening fraction and the heart rate (HR) corrected ejection time (Et_n_), namely:
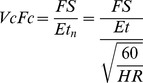

The ejection fraction was also calculated using the Simpson's rule (EF% simp), from a long axis view and four short axis views at different levels of the LV
The fractional area change (FAC%), was measured as
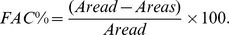

  First, we calculated the FAC% using one parasternal short-axis view at mid-ventricular level (FAC% short). Given the poor definition of the endocardial border on the short-axis view, especially at mid-ventricular and apical levels, caused by the apical location of the infarction in our model, we also calculated the FAC% in one EKV-mode parasternal long-axis view (FAC% long). Both short and long-axis views were acquired using gain settings that optimized the visualization of the endocardial and epicardial walls.
The peak velocity of the septal basal level (S′ septal) and the posterior wall (S′ post) were measured using Tissue Doppler from a four chamber and short axis view, respectively.
The cardiac output (CO), was measured from the left ventricular output tract length (LVOT) and HR measured on a long axis view, and the area under the curve of a Doppler measured on the aortic valve.

The LV diastolic function was evaluated using pulsed-wave Doppler sensing of the transmitral inflow obtained from LV apical two or four chamber views. From the pulsed-wave Doppler graph, we measured the peak E wave velocity (early filling wave), peak A wave velocity (late atrial contraction wave), E/A ratio, deceleration time (MVDT), ejection time from opening to closure of the aortic valve (ET ms) and LV isovolumetric relaxation (IVRT) and contraction (IVCT) times. During the scan, the ultrasound beam was kept as parallel as possible to the blood flow. Representative Doppler curves obtained from one of the animals in all time points are included as Figure S1.

The Tei Index –an indicator of global cardiac function- was calculated from the pulsed-wave Doppler graph:


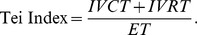


Finally, an estimate of both systolic and diastolic function was also obtained using tissue Doppler imaging on the LV septal basal portion of the mitral annulus, and on the LV posterior wall, taken from a parasternal short-axis view at the level of the papillary muscle. From the tissue Doppler imaging graph, we calculated the negative E′ wave (early diastolic myocardial relaxation velocity), negative A′ wave (late-atrial myocardial contraction velocity), and the ratio E/E′ (ratio of mitral peak velocity of early filling E to early diastolic mitral annular velocity E′) Representative tissue Doppler curves obtained from one of the animals in all time points are included as Figure S2.

All measurements were performed offline using dedicated Vevo770 quantification software (Vevo 770 v. 3.0.0).

### Tissue processing and staining

Mice were anesthetized, injected with 100 µl saline containing 0.1 mM cadmium chloride (Sigma), and perfusion-fixed for 10 min with 4% paraformaldehyde under physiological pressure. The hearts were excised, fixed overnight in 4% paraformaldehyde at 4°C, and cut in three 3.0 mm blocks (apical, mid-ventricular and basal) using a mouse heart slicer matrix (Zivic instruments, see Figure S3). Finally, hearts were dehydrated in 70% ethanol (4°C, o/n), embedded in paraffin and sectioned at 5 µm thickness. The sections were deparaffinated and stained by immersion in Picrosirius Red (Sigma) for 90 minutes, differentiated 2 minutes in HCl (Sigma) 0.01 N, dehydrated and mounted in DPX [27].

### Morphometric analysis

The histological extent of the infarction was measured as the amount of collagen deposition stained by Sirius red [22]. Briefly, an average of 15 serial sections per heart were imaged at 2.5× using a Zeiss Axio Imager M1 microscope (Carl Zeiss AG, Oberkochen, Germany) equipped with and AxioCam ICc3 digital camera and Axiovision software (v 4.6.3.0). The images were acquired as image mosaics mounted by the software that controls the microscope. The infarct size was automatically measured in each section photograph, using the AnalySIS^R^ software [29]. The results were calculated as percentage of infarcted area *vs.* total LV area and averaged through all the sections.

### Statistical analysis

All data are expressed as mean ± standard deviation. The Shapiro-Wilk test was used to verify that the data followed a normal distribution, which justified or not the use of a parametric test. A Student's *t*-test was used for the statistical analysis of means. To calculate the correlation between infarction size and FAC% we used the Pearson correlation and linear regression analysis. For all tests, a *P* value of less than 0.01 was considered statistically significant. All statistical analysis was performed using SPSS software for windows (Version 15.0).

## Results

### Echocardiographic data

The average study was 15 minutes long. Four mice from the experimental group were excluded from the study: Three of them died during surgery and one more 7 days after myocardial infarction. The results of the structural and functional measurements performed on the control –sham- group are included in Table S1. No significant differences between the basal and post-surgery (7 day) measurements were found in any of the measurements, thus the measurements obtained on the experimental group, summarized in Tables 1, 2, 3, 4, 5, and 6 can be considered free from artifacts caused by the surgery.

**Table 1:** Structural analysis.

Day	HR	Dbvi	Dmvi	Davi	Aread	Areas
**0**	364,15±27,20	3,09±0,38	3,36±0,20	2,82±0,24	18,98±1,81	6,87±0,93
**7**	366,54±39,49	3,27±0,35	3,63±0,30	3,49±0,42**	22,04±2,11**	13,81±2,80**
**14**	368,08±41,21	3,09±0,31	3,75±0,33*	3,26±0,32**	22,16±2,16**	14,31±2,48**
**30**	361,23±29,31	3,49±0,43	3,76±0,30**	3,60±0,41**	22,83±2,37**	14,69±2,75**
**60**	374,23±36,31	3,35±0,17	3,83±0,31**	3,48±0,28**	23,75±2,67**	15,30±3,16**
**90**	364,23±37,35	3,68±0,39**	3,95±0,37**	3,81±0,45**	24,08±2,45**	15,78±3,20**

**HR**: heart rate. **Dbvi**: LV diameter (basal). **Dmvi**: LV diameter (medium). **Davi**: LV diameter (apical). **Aread**: end-diastolic area. **Areas**: end-systolic area. (* indicates statistical significance versus day 0, with p<0.01) (** indicates statistical significance versus day 0, with p<0.001).

**Table 2:** Systolic function.

Day	EF% tei	EF% simp	FS%	VcFc	FAC% short	FAC% long
**0**	62,48±9,99	66,61±6,42	33,59±7,58	0,24±0,05	65,55±3,78	63,78±3,87
**7**	65,26±5,78	48,15±6,21**	35,43±4,75	0,25±0,05	47,89±3,99**	35,90±6,76**
**14**	60,76±5,56	51,69±4,59**	32,19±3,88	0,22±0,03	50,37±4,90**	35,69±7,08**
**30**	59,70±7,38	48,51±6,13**	31,59±5,19	0,21±0,04	49,96±3,74**	36,01±7,69**
**60**	63,11±3,45	45,76±10,50**	33,93±2,56	0,24±0,03	49,26±4,78**	36,16±6,74**
**90**	59,37±4,98	44,84±8,30**	31,31±3,44	0,22±0,03	46,78±3,87**	35,03±8,12**

**EF% tei**: ejection fraction using Teichholz. **EF% simp**: ejection fraction using Simpson's rule. **FS% tei**: fractional shortening using Teichholz. **VcFc**: normalized mean velocity of circumferential fiber shortening. **FAC% short**: fractional area change measured on a short axis view. **FAC% long**: fractional area change measured on a long axis view. (* indicates statistical significance versus day 0, with p<0.01) (** indicates statistical significance versus day 0, with p<0.001).

**Table 3:** Systolic function (cont'd).

Day	LVID d	LVID s	CO	S′ post	S′ septal
**0**	3,64±0,29	2,38±0,34	15,76±2,36	20,18±1,60	20,78±3,23
**7**	3,95±0,40	2,53±0,27	13,90±3,75	18,96±2,36	19,05±1,97
**14**	4,04±0,36*	2,72±0,31	15,40±4,20	18,96±1,79	17,91±1,23*
**30**	4,08±0,41*	2,80±0,43	17,18±4,95	18,81±1,31	17,41±1,09**
**60**	4,28±0,33*	2,85±0,32*	13,83±2,31	18,37±0,95*	17,74±2,70*
**90**	4,22±0,34**	2,90±0,28**	16,23±2,82	19,57±2,43	18,69±1,89

**LVID d**: LV internal diameter (diastole). **LVID s**: LV internal diameter (systole). **CO**: cardiac output. **S′ post**: peak velocity of the posterior wall. **S′ septal**: peak velocity of septal basal level. (* indicates statistical significance versus day 0, with p<0.01) (** indicates statistical significance versus day 0, with p<0.001).

**Table 4:** Diastolic function.

Day	E	A	E/A	ET ms	MVDT
**0**	670,38±69,46	410,55±49,86	1,58±0,23	57,79±5,36	21,25±1,48
**7**	614,52±78,08	317,09±73,61*	2,10±0,68	58,94±6,39	20,10±4,28
**14**	588,37±52,63*	411,86±88,69	1,63±0,46	58,73±5,74	19,43±1,97
**30**	558,02±74,43**	350,09±60,88	1,72±0,41	60,71±3,72	20,10±3,00
**60**	593,40±107,20	349,78±87,45	1,79±0,47	56,06±4,94	19,27±2,16
**90**	590,41±104,08	371,00±66,21	1,57±0,30	57,69±5,81	20,00±2,34

**E**: early filling wave. **A**: late atrial contraction wave. **E/A**: ratio between E and A. **ET ms**: ejection time from opening to closing of the aortic valve. **MVDT**: deceleration time. (* indicates statistical significance versus day 0, with p<0.01) (** indicates statistical significance versus day 0, with p<0.001).

**Table 5:** Diastolic function (cont'd).

Day	IVCT	IVRT
**0**	12,50±2,93	14,23±2,26
**7**	13,94±2,97	18,17±2,20**
**14**	14,33±3,81	18,27±3,25*
**30**	13,56±3,14	18,75±2,60**
**60**	12,31±2,54	17,98±2,82*
**90**	12,98±3,08	17,50±2,39*

**IVCT**: LV isovolumetric contraction time. **IVRT**: LV isovolumetric relaxation time. (* indicates statistical significance versus day 0, with p<0.01) (** indicates statistical significance versus day 0, with p<0.001).

**Table 6:** Global cardiac function.

Day	Tei index	E/E′	E′ septal	E′ post	A′ septal	A′ post
**0**	0,46±0,07	28,45±3,53	23,82±3,21	25,20±4,30	20,07±3,61	17,00±2,99
**7**	0,55±0,08*	29,59±6,68	21,51±4,52	22,13±3,76	18,03±3,46	17,20±3,49
**14**	0,56±0,08*	28,57±4,69	20,33±2,43*	22,08±3,81	17,07±3,66*	17,32±3,90
**30**	0,54±0,06*	27,86±3,90	20,23±2,71*	21,38±3,98	18,00±2,47*	17,45±4,67
**60**	0,54±0,08*	29,50±3,68	20,22±3,20*	22,11±3,87	19,09±4,19*	16,29±3,45
**90**	0,58±0,08*	29,29±4,53	20,44±3,66	21,83±3,44	20,44±3,33	17,39±3,11

**Tei index**: index tei. **E/E′**: ratio between E and E′. **E′ septal:** early diastolic myocardial relaxation velocity (septal level). **E′ post:** early diastolic myocardial relaxation velocity (posterior wall). **A′ septal:** late-atrial myocardial relaxation velocity (septal level). **A′ post:** late-atrial myocardial relaxation velocity (posterior wall). (* indicates statistical significance versus day 0, with p<0.01) (** indicates statistical significance versus day 0, with p<0.001).

### Measurement of left ventricular remodeling and cardiac function

#### LV structural analysis

Table 1 (HR) shows the average heart rate of the animals before MI and 7, 30 and 90 days post-infarction. There are no statistically significant changes between time points. Therefore no changes on LV-fractional area values can be attributed to changes in HR caused by non-properly controlled effects of the anesthetic.

Table 1 lists the LV diastolic average internal diameter at baseline (Dbvi), middle (Dmvi) and apical (Davi) levels before MI and 7, 30 and 90 days post-infarction. These values reveal progressive post-infarction remodeling of the left ventricle. Namely, at basal level (Dbvi), the LV diameter significantly increased (p<0.001) 90 days post-infarction. At middle level (Dmvi), a significant enlargement (p<0.01) was detected starting 14 days post-infarction and becoming prominent 30 days post-infarction (p<0.001). At apical level (Davi), a marked, statistically significant enlargement (p<0.001) appeared 7 days post-infarction that continued until the end of the study. Furthermore, the LV end-diastolic and systolic areas -Table 1 (Aread) & Table 1 (Areas)- increased significantly as well, starting 7 days post-infarction (p<0.001) and remained at that level for the rest of the study.

**Figure 1:**
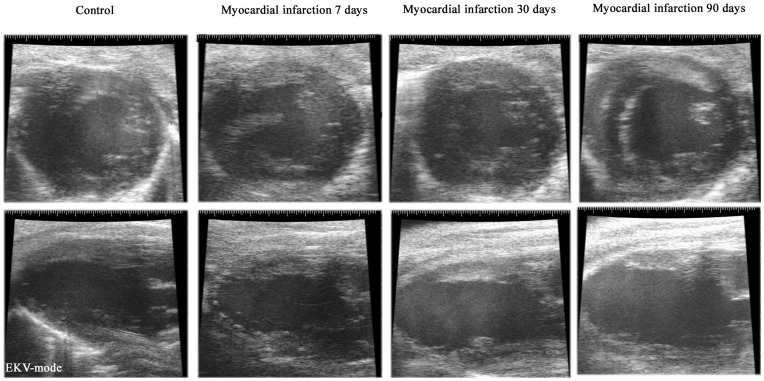
Visualization of the infarction. Sample EKV images at baseline and 7, 30 and 90 days post-infarction. Upper row: Parasternal short-axis views. Lower row: Parasternal long-axis views. The infarcted area is appreciated 7 days post-infarction and is located in the apical region. The evolution of the infarction results in progressive left ventricular remodeling. The videos from which the frames were selected are given as Supplementary material.

These data indicate that the ventricular dilation extends progressively from the apical origin of the infarction towards healthy myocardial areas, resulting in marked changes of both size and shape. This is clearly visualized in Figure 1, which presents sample EKV images of the LV of one of the animals at baseline and 7, 30 and 90 days post-infarction, both using a mid-level short-axis and a parasternal long-axis view. The corresponding videos are given as Videos S1, S2, S3, S4, S5, S6, S7, and S8. Due to the apical location of the origin of the infarction, the progression of the infarction, i.e. LV remodeling, is not clearly appreciated on the short-axis views (Videos S1, S2, S3, and S4), while it is evident in the long-axis views (Videos S5, S6, S7, and S8).

#### LV functional analysis

The systolic measurements taken on short axis views at the level of the papillary muscles -Table 2 (EF% tei, FS% & VcFc)- did not significantly change during the experiment. This is due –as already explained- to the apical origin of the infarctions in our model, and their slow progression towards the middle and baseline levels. That motivated the use of the Simpson's rule to calculate the EF% and the FAC measurements.

**Figure 2:**
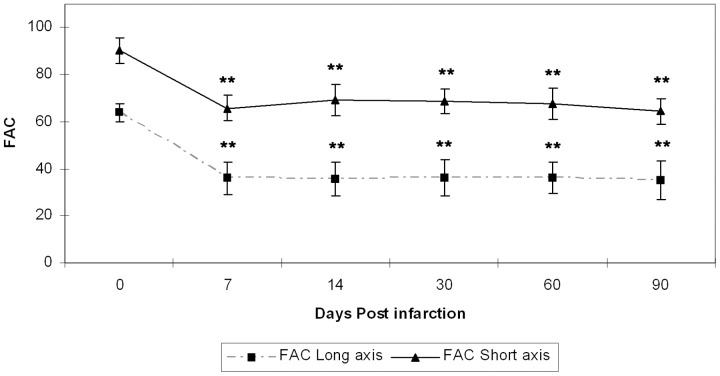
LV systolic function. FAC% measured both in parasternal short-axis views and a long-axis view. The FAC% decreased significantly as early as 7 days after myocardial infarction (** indicates statistically significance versus day 0, with p<0.001).

The EF% calculated using the Simpson's rule -Table 2 (EF% simp)- shows significant loss of systolic function starting 7 days post-infarction (p<0.001). Confirming this fact, Figure 2 illustrates the evolution of the LV systolic function using the FAC%. Namely, FAC% measured in a short axis view –Table 2 (FAC% short)- decreases significantly (p<0.001) 7 days post-infarction and remains virtually unchanged. This reduction of FAC% was more evident when measured in the parasternal long-axis view –Table 2 (FAC% long)-.

Finally, Table 3 (S′ septal & S′ post) contains the evolution of systolic function measured by tissue Doppler. The systolic tissue velocity S′ is degraded both at the septal basal level (S′ septal) and in the posterior wall (S′ post). This decrease is statistically significant between 14 and 60 days post-infarction when measured on the septal basal level (p<0.01), but it is only significant 60 days post-infarction when measured on the posterior wall (p = 0.01).

**Figure 3:**
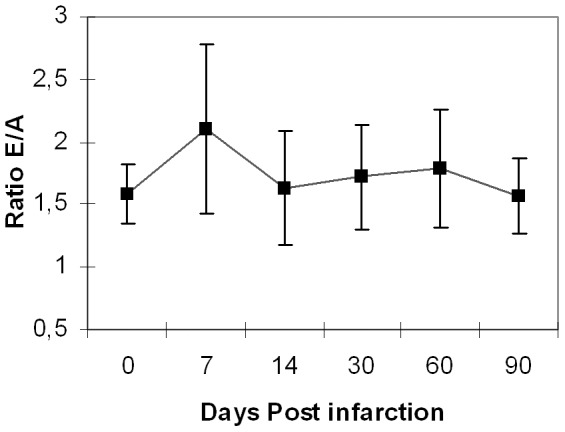
LV diastolic function. Evaluation of diastolic function measured as the E/A ratio calculated using the pulse Doppler wave mode at baseline and 7, 14, 30, 60 and 90 days post-infarction.

We used the pulsed wave Doppler of the mitral filling to measure diastolic dysfunction. We observed no significant increase of the ratio E/A -Table 4 (E/A) & Figure 3- and unchanged MVDT values (p = 0.405) during the entire duration of the study –Table 4 (MVDT)- . However, the IRVT increased significantly 7 days (p<0.001) post-infarction, and remained at that level until the end of the study (p<0.001), as seen in Table 5 (IVRT).

**Figure 4:**
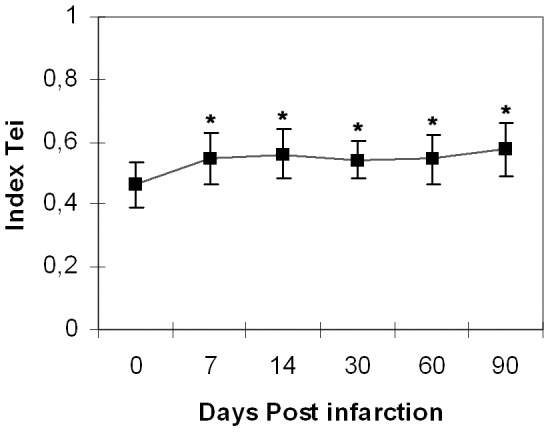
LV global cardiac function. Global cardiac dysfunction measured with the Tei Index at base line, 7, 14, 30, 60 and 90 days post-infarction. Changes are significant starting 7 days post-infarction until the end of the experiment (* indicates statistical significance versus day 0, with p<0.01).

The Tei Index, used as an indicator of global cardiac function, -Table 6 (Index Tei) & Figure 4- increased significantly 7 days post-infarction (p<0.01) and this increase was maintained until the end of the study (p<0.01).

The tissue Doppler analysis reveals significant deterioration of the diastolic function at the septal basal velocity –Table 6 (E′ septal)-, from 14 to 60 days post infarction (p<0.01), but no significant deterioration based on the posterior velocity –Table 6 (E′ post)-. Finally, the ratio E/E′ –Table 6 (E/E′)- did not present significant changes against the basal values any time during the study.

### Correlation between systolic functional changes and tissue remodeling

**Figure 5:**
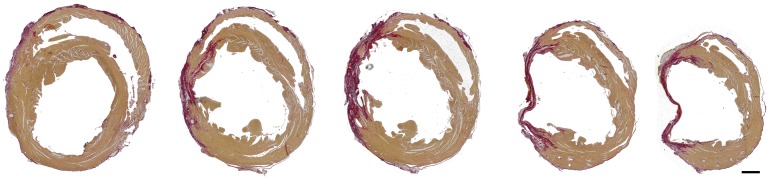
Histological analysis. Transverse sections of a heart 90 days after permanent ligation of the LAD. The sections were approximately taken starting at the level the papillary muscles (leftmost) to the apex (rightmost). The anterolateral myocardium is replaced by a thin fibrous scar tissue. The scale bar represents 1 mm.

At the end point of the experiment −90 days post-infarction-, the percentage of infarcted tissue was measured as described in the material and methods section. Figure 5 shows transverse sections of one sample heart, starting at the level of the papillary muscles (leftmost), and ending at the apex (rightmost). The muscle appears light brown-colored while the collagen in red, due to the Sirius red staining. Linear regression analyses demonstrated that the average infarct size measured from the histological sections correlated significantly with the FAC% values calculated in a long-axis view (r = −0.946; R^2^ = 0.90; p<0.05). However, the correlation with FAC% calculated in a short-axis view was lower (r = −0.812; R^2^ = 0.66), due to the difficulty of obtaining good short-axis views in this specific model.

## Discussion

The evaluation of cardiac function in mice has been hampered until this date by technical issues, such us the fast mouse heart rate, the difficulty to obtain clear echocardiographic views of the heart, and the translational motion present during image acquisition [16],[22],[23],[30]. In this study we present a complete structural and functional echocardiographic characterization of LV remodeling in a mouse model of long-term MI. To deal with the first two technical issues we used high-resolution ultrasound equipment dedicated for small animal imaging, which provide high-quality echocardiographic views of the LV of the mouse. In order to better visualize our apically located infarction, we have acquired and performed structural measurements in both parasternal short-axis and long-axis views. High resolution EKV mode images were used whenever necessary to improve the quality of the inherently noisy ultrasound images. Finally, the translational motion was partially compensated by the use of anesthetics.

This study has been carried out in the context of a broader effort aimed at characterizing the long-term effects of stem cell based therapies in post-infarction cardiac remodeling. We will use induced pluripotent stem (IPS) cells derived from DBA/2J cardiomyocytes, which will be injected in the infarcted myocardial areas. To avoid unwanted immunological response, the cells will be injected in infarcted hearts of DBA/2J mice. Previous studies on mouse models of MI were done mostly on C57BL mouse background [11],[19],[21],[30],[31],[32]. Since there is substantial evidence of different responses to MI due to age, sex or genetic background [10] we carried out a complete characterization of our DBA/2J MI based model. The common practice in these type of studies involves monitoring LV remodeling from 4 weeks to 9 weeks after surgery [11],[19], consistent with the general understanding that post-infarction complications can only be prevented very soon after the ischemic episode [27],[33] Instead, we monitor the cardiac function during 90 days after LAD ligation because that is the prospective duration of our cell-based therapy.

We have done a complete characterization of our myocardial infarction model by measuring the untreated progression of the LV systolic, LV diastolic and global cardiac function post MI. Other groups have done similar studies using the same equipment [34],[35],[36], but only used standard measurements. In our model, given the location of the infarction –starting at the ventricular apex and progressing towards the middle ventricle- we have measured the FAC% not only using the standard short-axis views but also in a parasternal long-axis view. Both results reflect marked systolic dysfunction caused by LV remodeling. However, the correlation between FAC% and infarct size estimated from the histology was higher for the FAC% measured in a single long-axis view than when measured in the short-axis views. This is due to the difficulty to obtain LV short-axis views with a clear definition of the entire post-infarction endocardial border, because of the orientation of the heart and the small size of the rib cage [23].

Our analysis of the evolution of FAC% with time shows that systolic dysfunction –visible as early as 7 days after MI- is directly related to the progressive dilation of the LV internal diameter and with the increase of the end-systolic and end-diastolic areas. However, changes in the diastolic function were not so evident, and were only detected using the IVRT. Contrarily, we found no significant changes of DT and the ratio E/A, while E′ measured at the posterior wall reflected significant changes only 14 days post-infarction. This is consistent with the findings of recent studies that claim that the isolated analysis of systolic or diastolic function may not be reflective of overall cardiac function [24],[25]. Moreover, it is well know that the common parameters of systolic and diastolic function are influenced by the anesthesia used for the mouse immobilization [22],[23]. Therefore, we also computed the combined myocardial performance Tei Index, which is independent of the heart rate or blood pressure levels. Our measurements confirmed significant impairment of global cardiac function starting 7 days post-infarction.

The measurement of the infarcted area in tissue sections is the common ex-vivo approach to determine the infarct size in animal models [11]. A recent study estimated the infarct size in vivo by calculating the infarct area from sequential B-mode echocardiographic short-axis images in a mouse model of MI, demonstrating significant correlation between the estimated infarct size and the EF% [8]. Takagawa *et al.* [11] reported that infarct size derived from area, length and midline length measurements all reflect the severity of systolic dysfunction. In that line, our linear regression analysis also finds significant correlation between long-axis view FAC% and the histological infarcted size measured as the mean percentage of infarcted area vs. total LV area.

In summary, we have performed a complete characterization of LV remodeling in a mouse model of MI, establishing a valuable control for future pharmacological, cellular or tissue engineering studies of post-infarction cardiac remodeling, such as those reported by us and others [27],[29],[33],[34],[35],[36].

## Supporting Information

Figure S1 **Pulsed-wave Doppler analysis.** Sample Doppler pulsed-wave recordings of the transmitral inflow obtained from LV apical two or four chamber views. The recordings were obtained from the same animal before infarction (top) and 7, 14, 30, 60 and 90 days (bottom) after infarction.
(TIF)

Figure S2 **Tissue Doppler analysis.** Sample tissue Doppler recordings of the LV septal basal portion of the mitral annulus, taken from a parasternal short-axis view at the level of the papillary muscle. The recordings were obtained from the same animal before infarction (top) and 7, 14, 30, 60 and 90 days (bottom) after infarction.
(TIF)

Figure S3 **Tissue processing.** A) Rodent heart slicer (Zivic Instruments). The heart is located into the slicer hole and two blades (Stanley) inserted (3 mm separated of each other). Once the blades are partially inserted, they are aligned with another one and simultaneously pressed down until the end. B) Heart blocks. The blades are raised out and the three heart blocks (apical, mid-ventricular and basal) removed.
(TIF)

Table S1 **HR**: heart rate. **EF% tei**: ejection fraction using Teichholz. **EF% simp**: ejection fraction using Simpson's rule. **FS% tei**: shortening fraction using Teichholz. **FAC% short**: fractional area change measured on a short axis view. **FAC% long**: fractional area change measured on a long axis view. **VcFc**: normalized mean velocity of circumferential fiber shortening. **LVID d**: LV internal diameter (diastole). **LVID s**: LV internal diameter (systole). **CO**: cardiac output. **LV vol d**: LV volume (diastole). **LV vol s**: LV volume (systole) (* indicates statistical significance versus day 0, with p<0.01) (** indicates statistical significance versus day 0, with p<0.001)
(DOCX)

Video S1 **Parasternal short axis sample EKV video recording at baseline.**
(AVI)

Video S2 **Parasternal short axis sample EKV video recording 7 days post-infarction.**
(AVI)

Video S3 **Parasternal short axis sample EKV video recording 30 days post-infarction.**
(AVI)

Video S4 **Parasternal short axis sample EKV video recording 90 days post-infarction.**
(AVI)

Video S5 **Parasternal long axis sample EKV video recording at baseline.**
(AVI)

Video S6 **Parasternal long axis sample EKV video recording 7 days post-infarction.**
(AVI)

Video S7 **Parasternal long axis sample EKV video recording 30 days post-infarction.**
(AVI)

Video S8 **Parasternal long axis sample EKV video recording 90 days post-infarction.**
(AVI)
