# Supplementary figures and images for: New Strategies for Echocardiographic Evaluation of Left Ventricular Function in a Mouse Model of Long-Term Myocardial Infarction
Source: PLoS One. 2012 Jul 27;7(7):e41691. doi: 10.1371/journal.pone.0041691 (PMC3407217; doi:10.1371/journal.pone.0041691)

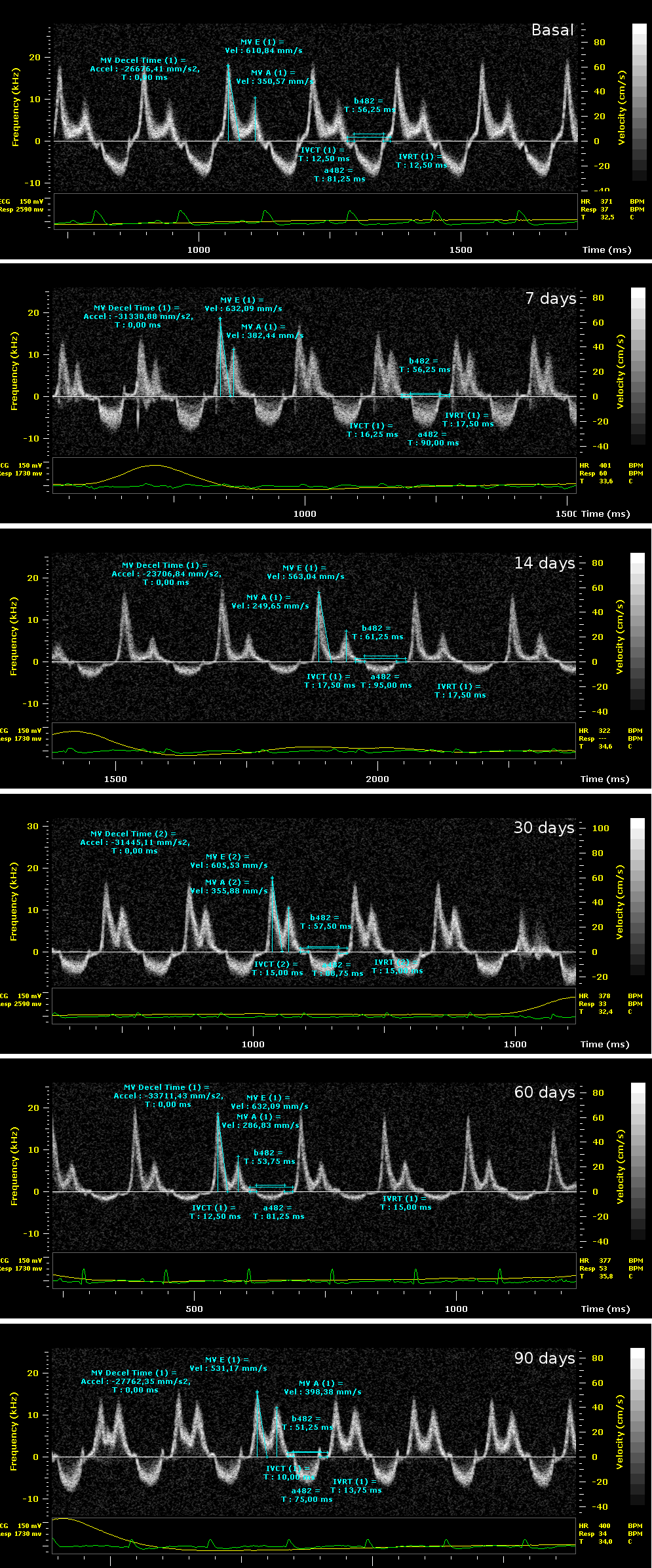

Supplement: Figure S1 — Pulsed-wave Doppler analysis. Sample Doppler pulsed-wave recordings of the transmitral inflow obtained from LV apical two or four chamber views. The recordings were obtained from the same animal before infarction (top) and 7, 14, 30, 60 and 90 days (bottom) after infarction. (TIF) [file pone.0041691.s001.tif]

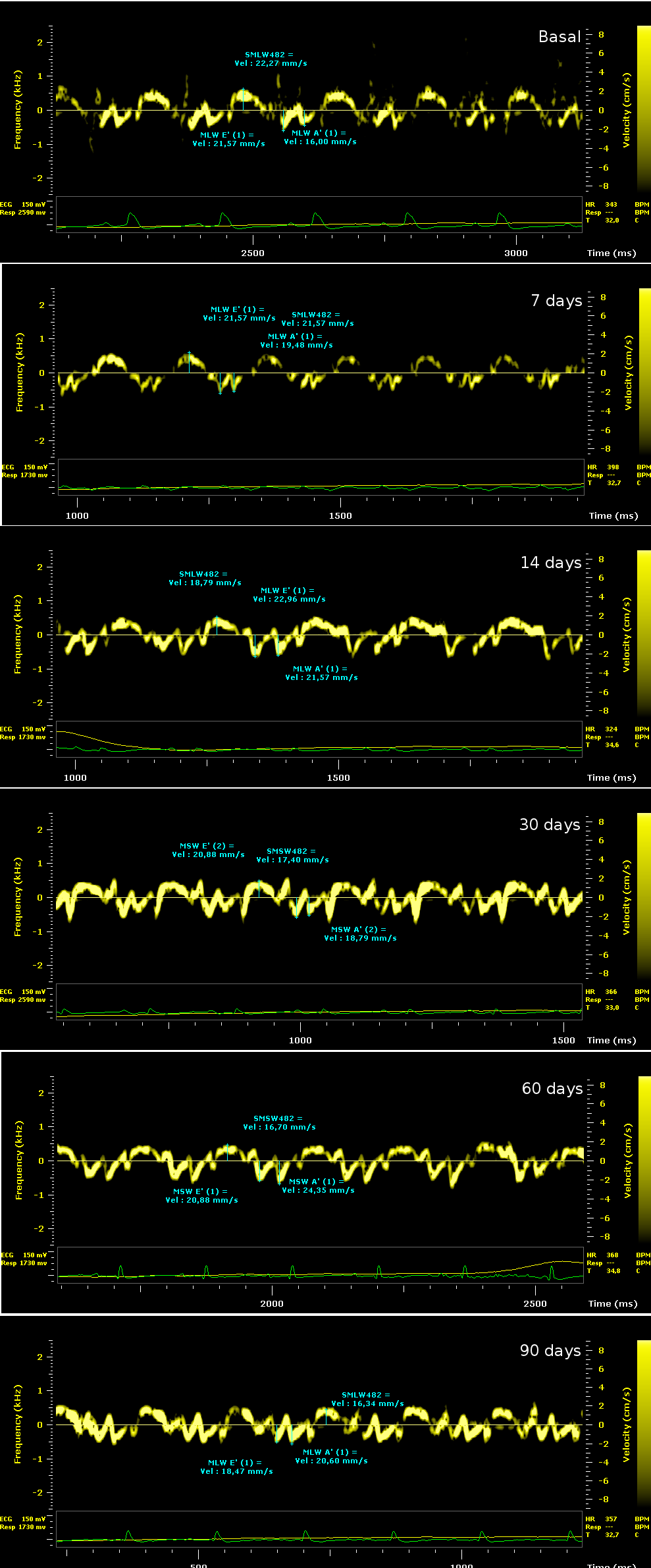

Supplement: Figure S2 — Tissue Doppler analysis. Sample tissue Doppler recordings of the LV septal basal portion of the mitral annulus, taken from a parasternal short-axis view at the level of the papillary muscle. The recordings were obtained from the same animal before infarction (top) and 7, 14, 30, 60 and 90 days (bottom) after infarction. (TIF) [file pone.0041691.s002.tif]

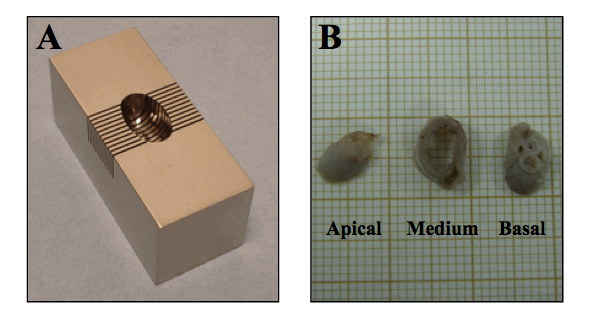

Supplement: Figure S3 — Tissue processing. A) Rodent heart slicer (Zivic Instruments). The heart is located into the slicer hole and two blades (Stanley) inserted (3 mm separated of each other). Once the blades are partially inserted, they are aligned with another one and simultaneously pressed down until the end. B) Heart blocks. The blades are raised out and the three heart blocks (apical, mid-ventricular and basal) removed. (TIF) [file pone.0041691.s003.tif]
