# Supplementary material for: New Strategies for Echocardiographic Evaluation of Left Ventricular Function in a Mouse Model of Long-Term Myocardial Infarction
Source: PLoS One. 2012 Jul 27;7(7):e41691. doi: 10.1371/journal.pone.0041691 (PMC3407217; doi:10.1371/journal.pone.0041691)
Supplement: Table S1 — HR: heart rate. EF% tei: ejection fraction using Teichholz. EF% simp: ejection fraction using Simpson's rule. FS% tei: shortening fraction using Teichholz. FAC% short: fractional area change measured on a short axis view. FAC% long: fractional area change measured on a long axis view. VcFc: normalized mean velocity of circumferential fiber shortening. LVID d: LV internal diameter (diastole). LVID s: LV internal diameter (systole). CO: cardiac output. LV vol d: LV volume (diastole). LV vol s: LV volume (systole) (* indicates statistical significance versus day 0, with p<0.01) (** indicates statistical significance versus day 0, with p<0.001) (DOCX) [file pone.0041691.s004.docx]

**SUPPLEMENTARY TABLES**

| **Day** | **HR** | **EF % tei** | **EF% simp** | **FS%** | **FAC% short** | **FAC% long** |
| --- | --- | --- | --- | --- | --- | --- |
| **0** | 347,10 ± 22,25 | 63,92 ± 3,34 | 63,59 ± 2,33 | 34,10 ± 2,36 | 59,34 ± 3,01 | 64,48 ± 3,96 |
| **7** | 347,37 ± 27,16 | 63,69 ± 3,07 | 63,02 ± 2,18 | 33,90 ± 2,04 | 59,22 ± 2,44 | 62,08 ± 8,92 |
| **Supplementary Table 1. Parameters of the control –sham- group** | | | | | | |

| **Day** | **VcFc** | **LVID d** | **LVID s** | **CO** | **LV vol d** | **LV vol s** |
| --- | --- | --- | --- | --- | --- | --- |
| **0** | 0,22 ± 0,02 | 3,64 ± 0,33 | 2,40 ± 0,26 | 15,88 ± 1,41 | 56,70 ± 11,71 | 19,90 ± 5,35 |
| **7** | 0,23 ± 0,03 | 3,58 ± 0,31 | 2,37 ± 0,25 | 16,69 ± 1,25 | 52,11 ± 14,43 | 19,86 ± 5,24 |
| **Supplementary Table 1 (cont’d)** | | | | | | |

Supplementary Table 1. **HR**: hear rate. **EF% tei**: ejection fraction using Teichholz. **EF% simp**: ejection fraction using Simpson’s rule. **FS% tei**: shortening fraction using Teichholz. **FAC% short**: fractional area change measured on a short axis view. **FAC% long**: fractional area change measured on a long axis view. **VcFc**: normalized mean velocity of circumferential fiber shortening. **LVID d**: LV internal diameter (diastole). **LVID s**: LV internal diameter (systole). **CO**: cardiac output. **LV vol d**: LV volume (diastole). **LV vol s**: LV volume (systole) (* indicates statistical significance with p<0.01) (** indicates statistical significance with p<0.001)
